# Noninvasive Assessment of Urinary Exfoliated Proximal Tubule Cell Multispectral Autofluorescence May Differentiate between Causes of Kidney Transplant Dysfunction

**DOI:** 10.34067/KID.0000000879

**Published:** 2025-06-20

**Authors:** Henry H.L. Wu, Yandong Lang, Shannon Handley, Aline Knab, Adnan Agha, Yuan Tian, Akanksha Bhargava, Ewa M. Goldys, Carol A. Pollock, Sonia Saad

**Affiliations:** 1 Renal Research Laboratory, Kolling Institute of Medical Research, Royal North Shore Hospital, The University of Sydney, Sydney, New South Wales, Australia; 2 ARC Centre of Excellence for Nanoscale Biophotonics, School of Biomedical Engineering, The University of New South Wales, Sydney, New South Wales, Australia; 3 Department of Renal Medicine, Royal North Shore Hospital, Sydney, New South Wales, Australia

**Keywords:** acute rejection, chronic rejection, histopathology, kidney transplantation, proximal tubule, rejection, renal fibrosis, renal proximal tubule cell, artificial intelligence

## Abstract

**Key Points:**

- There is an unmet critical need currently for a noninvasive approach to accurately diagnose the cause of kidney transplant complications.
- Cell multispectral autofluorescence signals have demonstrated to be highly biologically informative, reflecting cell and tissue metabolic status.
- Urinary exfoliated proximal tubule cell multispectral autofluorescence could potentially be used to differentiate between causes of transplant graft dysfunction.

**Background:**

Complications relating to delayed or deteriorating graft function following kidney transplantation are common. There is no validated method apart from transplant kidney biopsy which can accurately identify between the histopathologic causes of graft dysfunction. Considering an unmet critical need for a noninvasive approach to reliably diagnose kidney transplant complications, this work proposes a novel methodology based on the assessment of exfoliated proximal tubule cells (PTCs) extracted from urine of kidney transplant recipients by using their multispectral autofluorescence features.

**Methods:**

Three groups of ten patients who have undergone clinically indicated transplant kidney biopsy and was subsequently diagnosed with either acute tubular necrosis (ATN), graft rejection or non–rejection-associated interstitial fibrosis, and tubular atrophy (IFTA) took part in this study. Exfoliated PTCs from urine collected before transplant biopsy were extracted using a validated immunomagnetic separation method based on anti-CD13 and anti-sodium-glucose co-transport 2 antibodies. Imaging was performed on a custom-made multispectral autofluorescence microscopy and camera system. Multispectral autofluorescence images of PTCs were quantitatively analyzed by using optimized small sets of features to prevent overfitting. Binary classification was performed by a random forest classifier and the AutoGluon machine learning software. The results were validated by five-fold cross-validation.

**Results:**

For random forest classification, features were selected using entropy-based feature selection, resulting in area under the curve values of 0.92 (ATN versus graft rejection), 0.86 (ATN versus IFTA), and 0.62 (graft rejection versus IFTA) respectively. The AutoGluon classifier optimisation for the same features resulted in area under the curve values of 0.95 (ATN versus graft rejection), 0.92 (ATN versus IFTA), and 0.91 (graft rejection versus IFTA).

**Conclusions:**

Our results demonstrate a proof-of-concept that measurement of autofluorescent features from urinary exfoliated PTCs multispectral autofluorescence could differentiate between patient groups with ATN, graft rejection, and IFTA in kidney transplant recipients to an excellent degree of accuracy using AutoGluon classifier optimisation.

## Visual Abstract

**Figure:**
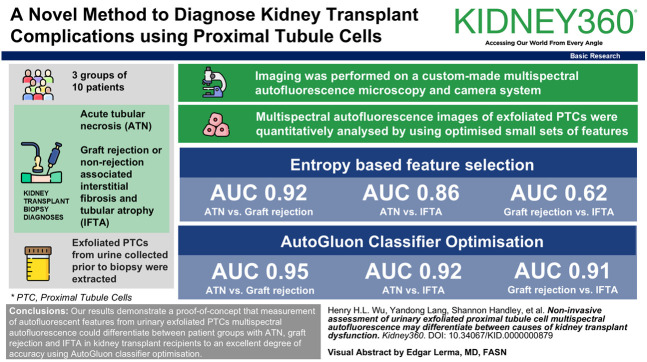

## Introduction

CKD is a progressive condition which may potentially result in kidney failure, where patients require KRT in the form of dialysis or kidney transplantation.^1,2^ Although kidney transplantation is often the preferred option of treatment for kidney failure, optimal outcomes after kidney transplantation may be challenged due to various complications that can be defined histologically.^3–6^ Complications such as acute tubular necrosis (ATN) and acute rejection can lead to delayed graft function in the acute phase after transplantation, whereas in the longer term, the transplanted kidney may also experience progressive dysfunction due to progressive kidney interstitial fibrosis and tubular atrophy (IFTA) from a variety of etiologies not limited to chronic allograft nephropathy, calcineurin inhibitor toxicity, chronic rejection, and progressive kidney fibrosis in the absence of immunologic challenge or recurrence of rejection episode(s).^5,6^

Current epidemiologic data suggest that delayed or deteriorating graft function occurs in between 20% and 50% of kidney transplant cases.^5^ It is particularly important to positively identify between the histopathology of those who have delayed or deteriorating graft function, as the treatment strategy for the various causes of graft dysfunction would be different.^7^ There is no strongly validated method apart from biopsy which can definitively predict the cause of delayed or deteriorating graft function at present. Transplant biopsy is invasive, requires hospitalization, is time consuming, and is costly.^8–10^ Hence, there is an unmet critical need for accurate noninvasive diagnostic approaches to identify kidney transplant complications.^11^

The proximal tubule cells (PTCs) make up over 50% of the kidney mass.^12^ Dysfunction of PTCs is related to the pathogenesis of kidney transplant dysfunction.^13,14^ This suggests that examination of PTCs can have potential diagnostic and prognostic value in patients with delayed or deteriorating graft function. Kidney tubules are continuously exposed to glomerular filtrate, and thousands of living PTCs are excreted daily in the urine together with other cells shed from different parts of the nephron, ureters, bladder, and urethra.^15,16^ Viable exfoliated PTCs can be isolated from urinary sediment.^17,18^ To date, there remains no sensitive method to determine if exfoliated PTCs can be used to diagnose and differentiate between patients with different histopathologic causes of graft dysfunction after kidney transplantation.

Our group developed a novel methodology based on multispectral assessment of native cell autofluorescence—the color of the cells' “natural glow”—which has been specifically shown to sensitively detect metabolic changes and oxidative stress at a cellular level.^19,20^ Cell autofluorescence is derived from native fluorophores such as collagen, elastin, tryptophan, reduced nicotinamide adenine dinucleotide (phosphate), and flavins, which play a pivotal role in cell and tissue structure, as well as cell and tissue metabolism.^21,22^ This native autofluorescence can be collected using multispectral microscopy which captures images with various emission and excitation wavelength ranges, yielding spectrally dependent quantitative features for each individual cell, including parameters such as average channel intensities, channel intensity ratios, pixel SDs, and skewness.^23,24^ Such data provide each cell with a unique signature, which originates from its intracellular biochemistry and organization. Past studies have noted that these cellular characteristics may be highly biologically informative and can distinguish biologic conditions, including cell cycle stage,^19^ cellular inflammation,^25^ level of reactive oxygen species,^20^ presence of neoplasia,^26^ and cell aging^27^ through assessment of cell autofluorescence. As these are all key pathophysiologic factors to consider in kidney disease, we evaluated whether multispectral autofluorescence features in urinary exfoliated PTCs can be applied to differentiate between individuals with different histopathologic causes of kidney transplant dysfunction.^28^

## Methods

**Figure 1:**
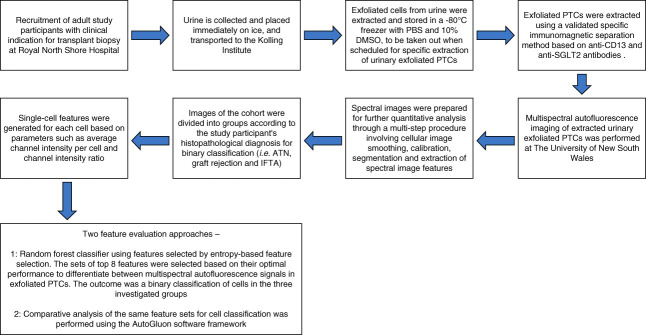
**Workflow flow chart summarizing key aspects of the study methodology.** ATN, acute tubular necrosis; DMSO, dimethyl sulfoxide; IFTA, interstitial fibrosis and tubular atrophy; PTC, proximal tubule cell; SGLT2, sodium-glucose co-transport 2.

A summary workflow flow chart describing the key aspects of our study methodology is illustrated in Figure 1.

### Study Participant Recruitment and Ethical Considerations

The study included randomly selected adult individuals of either sex aged between 18 and 75 years under the care of Royal North Shore Hospital with previous kidney transplantation(s) and with a clinically indicated transplant kidney biopsy (either as a protocol or as-needed biopsy) performed immediately after prebiopsy urine sample collection. Collection of human data was approved by the human ethics committee at Royal North Shore Hospital (Ref: HREC/17/HAWKE/471) and the University of New South Wales, Sydney, Australia (Ref: HC180710). Data collection in this study was performed in accordance with relevant local guidelines and regulations. The clinical and research activities being reported from our center are consistent with the Principles of the Declaration of Istanbul as outlined in the “Declaration of Istanbul on Organ Trafficking and Transplant Tourism.” This study adheres to the Declaration of Helsinki, and informed consent was obtained from all study participants.

### Procurement of Allograft Kidney Biopsy Tissue

The procurement of allograft kidney biopsy tissue was performed at Royal North Shore Hospital. Before commencing the procedure, written informed consent was obtained from study participants to collect the prebiopsy urine sample for the purposes of the study and to obtain the allograft kidney biopsy tissue. Allograft kidney biopsy was then performed in an ultrasound-guided manner using a sterile technique. This was repeated two or three times depending on whether an adequate sample, subject to the operator's evaluation, was obtained. A dressing was then applied to the site of needle insertion, and a further ultrasound scan was performed to check for any immediately occurring complications. Patients were required to lie flat for 4 hours with a further 1–2 hours sitting on bed. They were monitored by nursing staff during this period for hemodynamic stability before discharge.

### Evaluation of Allograft Kidney Biopsy Tissue to Determine Study Participant Groups

Tissue obtained from allograft biopsies was subsequently transferred to the histopathology department at Royal North Shore Hospital and urgently assessed as per standard protocol—with the majority of the sample processed for light microscopic evaluation *via* paraffin-embedded sections, supplemented by special and immune histochemical stains, and some reserved for immunofluorescence and electron microscopic studies if indicated. Light microscopy assessment included a minimum of two hematoxylin and eosin, two periodic acid–Schiff, two Masson trichrome (trichrome), and two Jones methenamine silver (silver) stains in complementary fashion. Hematoxylin and eosin stains provided a general overview of all structures; cytoplasmic and nuclear features; periodic acid–Schiff stains highlighted tubular and glomerular basement membranes; trichrome stains accentuated fibrous tissue and fibrin, if present; and silver stains highlighted the glomerular and tubular basement membranes and also sclerosis. An immune histochemical or immunofluorescence stain for C4d was also routinely used to evaluate for antibody-mediated rejection. The biopsy assessment was conducted and reported by accreditation-qualified pathologist assessors in New South Wales Health Pathology Laboratory, Department of Anatomical Pathology, Northern Sydney Local Health District, Sydney, Australia.

Study participants were divided into three groups, based on findings from their allograft biopsy detailing the likely cause of delayed or deteriorating graft function. Our study aimed to differentiate between patients reported with ATN, graft rejection, or non–rejection-associated IFTA (simply termed as IFTA hereon) in transplant biopsy by binary classification using multispectral assessment of cell autofluorescence.

### Demographic and Clinical Characteristics of Study Participant Groups

Study participants' demographic, alongside clinical and biochemical data were acquired from the Royal North Shore Hospital electronic medical records, summarized using appropriate descriptive statistics and compared between the three groups. For variables with symmetric normal distributions, the mean and SD were reported. For variables that were skewed or ordinal, the median and interquartile range were used for summarization. Proportions were also presented for categorical variables. Continuous variables between the groups were compared using the ANOVA test (if normally distributed) or the Kruskal-Wallis test (if the distribution was not normal). Categorical variables were compared using the chi-squared test or Freeman-Halton extension of the Fisher exact test accounting for sparsely distributed data.

A total of 30 study participants were included, including ten individuals with ATN, ten individuals with graft rejection and ten individuals with IFTA. Supplemental Table 1 presents the demographic and clinical characteristics for individual study participants between the three groups. Table 1 presents the overall cohort profile and compares demographic and clinical characteristics between the three groups. The mean age in the ATN and IFTA groups are significantly older compared with the graft rejection group (55.0 [ATN] versus 39.2 [rejection] versus 54.1 years [IFTA], *P* = 0.028), while the median number of days between kidney transplantation and allograft biopsy was significantly much higher in the IFTA group compared with the other two groups (5 [ATN] versus 96 [rejection] versus 1939 days [IFTA], *P* < 0.001), as expected. Otherwise, there were no statistically significant differences in the demographic and clinical characteristics between the groups.

**Table 1:** Baseline demographic and clinical characteristics of the study groups (*n*=30)

Characteristic	ATN (*n*=10)	Rejection (*n*=10)	IFTA (*n*=10)	*P* Value^a^
Sex (male), *n* (%)	7 (70)	8 (80)	5 (50)	0.350
Age (yr), mean (SD)	55.0 (8.4)	39.1 (9.4)	54.1 (13.2)	0.028^b^
No. of days from transplant to biopsy, median (IQR)	5.0 (2.5)	96 (137.5)	1939 (1508.5)	<0.001^b^
eGFR (ml/min per 1.73 m^2^), median (IQR)	33.5 (20)	34.5 (9.8)	26.5 (6.5)	0.261
uACR, median (IQR)	45 (20)	37 (31.8)	82 (74)	0.112
Albuminuria, *n* (%)	Micro: 8 (80) Macro: 2 (20)	Micro: 9 (90) Macro: 1 (10)	Micro: 5 (50) Macro: 5 (50)	0.110
Serum creatinine (*µ*mol/L), median (IQR)	179.5 (160.3)	196 (27.8)	238 (91)	0.814
Distribution of allograft biopsy diagnosis, *n* (%)	All ATN	Acute ABMR: 2 (20) Acute TCR: 1 (10) Chronic active ABMR: 2 (20) Recurrent ABMR: 1 (10) Chronic TCR: 2 (20) Recurrent TCR: 1 (10) Mixed chronic BCR and TCR: 1 (10)	Chronic allograft nephropathy: 2 (20) CNI toxicity-associated IFTA: 4 (40) Diabetic nephropathy: 1 (10) Progressive fibrosis in the absence of immunologic challenge: 3 (30)	N/A

ABMR, antibody-mediated rejection; ACR, albumin-creatinine ratio; ATN, acute tubular necrosis; BCR, B-cell rejection; CNI, calcineurin inhibitor; IFTA, interstitial fibrosis and tubular atrophy; IQR, interquartile range; N/A, not applicable; TCR, T-cell rejection.

a *P* values are calculated using the ANOVA test (if normally distributed) or Kruskal-Wallis test (if the distribution is nonparametric) for continuous variables, and the chi-squared test or Freeman-Halton extension of the Fisher exact test accounting for sparsely distributed data for categorical variables.

b Statistical significance with *P* < 0.05.

### Collection of Urine Samples and Extraction of Urinary Exfoliated PTCs

Using urine bottles with capacity of up to 200 ml, spot urine samples were collected from study participants for each study participant. Each collected urine sample was placed on ice immediately after collection for transportation to the Renal Research Laboratory, Kolling Institute of Medical Research, and was centrifuged for 20 minutes at 4°C to collect exfoliated cells and then washed twice with PBS. Before extraction of PTCs, the initially processed sample were stored at −80°C with PBS with 10% dimethyl sulfoxide.^28^ Specific PTCs expressing CD13, sodium-glucose co-transport 2, and angiotensinogen were extracted through a validated specific immunomagnetic separation methodology we have previously described.^28^ Extracted cells were subsequently resuspended in 200μl of PBS on a 13-mm glass bottom petri dish (Cell E&G, and #GDB0004-200) and viewed in brightfield microscopy. Quantification of the number of exfoliated PTCs in brightfield microscopy was performed by H.H.L. Wu for the entirety of this experiment.

### Multispectral Autofluorescence Imaging of Extracted Urinary Exfoliated PTCs from Allograft Kidneys

Multispectral autofluorescence imaging was conducted using a custom-made autofluorescence microscopy system, built by adapting a standard fluorescence microscope (Olympus iX83).^22,23,29^ The light source (from Quantitative Pty Ltd, AU) in this single photon autofluorescence microscope provides a number of defined narrowband (±5 nm) excitation wavelength ranges through low power light-emitting diodes and several epifluorescence filter cubes producing defined spectral channels (34 channels). The channels span wide excitation (340–510 nm) and emission (420–650 nm) wavelength ranges (Supplemental Table 2 presents the details of the spectral channels). A 40× oil (nicotinamide adenine dinucleotide 1.15) objective, with transmission in the ultraviolet range, was used for imaging. All images were captured by a Nüvü electron-multiplying charge-coupled device camera HNü 1024 cooled to −65°C to reduce sensor-induced noise in the images (1024×1024 pixels). Image acquisition times of 5 seconds per channel with three times averaging was applied to optimize image quality (*i.e*., signal-to-noise ratio). A sequence of fluorescence images for each spectral channel was collected for each sample area, resulting in a “data block.” Each data block is supplemented by a brightfield image showing cell morphology. Representative brightfield and multispectral channel cell images from each group are presented in Supplemental Figure 1. In addition to cell imaging, reference images were captured of water as background fluorescence and calibration fluid (30 *µ*M nicotinamide adenine dinucleotide mixed with 18 *µ*M flavin adenine dinucleotide) displaying fluorescence across all channels. The calibration fluid was additionally measured on a calibrated spectrofluorometer (Flouromax 4) providing reference values. Manual cell segmentation was performed on brightfield images, generating masks outlining the PTCs.

### Cellular Image Preparation and Feature Extraction for Analysis

After image acquisition, the spectral images were prepared for quantitative analysis applying image smoothing to reduce the impact of noise, subtracting background autofluorescence, and implementing a calibration method.^30^ Image smoothing minimized the impact of Poisson's noise and dead or saturated pixels. The calibration mechanism involved eliminating background fluorescence, adjusting for uneven illumination of the field of view, and aligning the multispectral images with reference values form the Fluoromax 4 as reported in previous works by our group.^21,30^ PTCs in the images were manually segmented.^31^ After image preparation and segmentation, image features across all channels were computed for each segmented cell. A total of 7703 hand-crafted, quantitative features per cell were generated using the Python programming software. The features included cell-average intensities in each channel and channel intensity ratios.

### Machine Learning Analysis to Differentiate between Multispectral Autofluorescence Signals in Exfoliated PTCs between Individuals across the Study Groups

Binary classification of cells from different groups of patients was performed using two different methods: method 1, a random forest classifier, and method 2, by applying the AutoGluon machine learning software from Amazon Web Services to explore and rank alternative classifiers. In each of these methods, small feature sets were selected to avoid overfitting. In method 1, entropy-based feature selection was used to yield different sets of optimal eight features (Supplemental Table 3) to compare between the groups ATN versus graft rejection, ATN versus IFTA, and graft rejection versus IFTA. In method 2, we used AutoGluon.Tabular (version: 0.8.3b20231023), specifically employing the TabularDataset module for tabular data preprocessing and the TabularPredictor module for model training and prediction.^32^ These modules were used to explore alternative classifiers available in this software on the same feature combinations from Supplemental Table 3 for each of the defined group comparisons. Detailed instructions relating to AutoGluon can be found at this link (https://auto.gluon.ai/stable/tutorials/tabular/tabular-quick-start.html). In methods 1 and 2, five-fold cross-validation was conducted to validate our results. This is a process where analyzed cells are divided into a training set and testing set in five different ways. The area under the curve (AUC) results are then averaged, and the variance is calculated. This ensures that there is no inherent bias in developing an analysis model based on the investigated patient cohort. Performance metrics are defined by AUC values (and the error bars representing 95% confidence intervals) in the receiver operating characteristic curves.

## Results

Multispectral autofluorescence imaging was completed for 161 cells in total (51 cells in the ATN group; 60 cells in the graft rejection group; 50 cells in the IFTA group).

### Differentiation of Multispectral Autofluorescence Signals in Exfoliated PTCs between Study Participants with ATN and Graft Rejection

**Figure 2:**
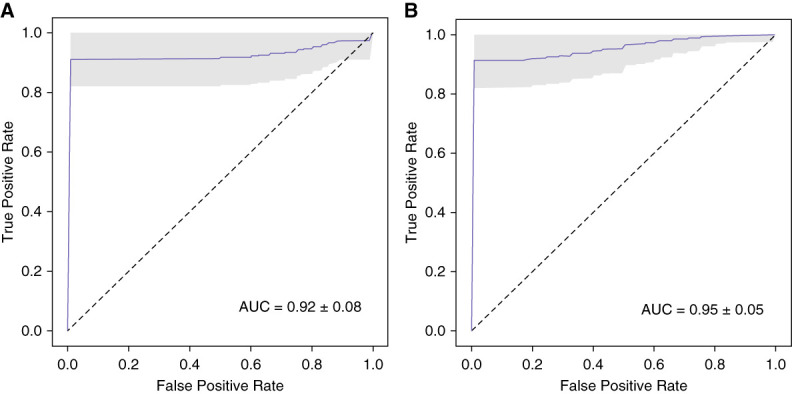
**Multispectral differentiation of exfoliated PTCs between study participants with ATN or graft rejection.** (A) ROC curve for our obtained cell classifier using the optimal spectral feature combination selected by method 1. (B) ROC curve obtained using the AutoGluon classifier framework for comparative analysis (*i.e*., method 2) with the same feature combination which was selected by method 1. AUC, area under the curve; ROC, receiver operating characteristic.

On selecting their optimal feature combination (Supplemental Table 3) using method 1, we were able to categorise PTCs between these two groups with an AUC value of 0.92±0.08 (Figure 2A). The analysis using AutoGluon in method 2 and the same feature combination revealed significant differences in multispectral cellular features between exfoliated PTCs from study participants in these groups with an AUC value of 0.95±0.05 (Figure 2B).

### Differentiation of Multispectral Autofluorescence Signals in Exfoliated PTCs between Study Participants from ATN and IFTA Groups

**Figure 3:**
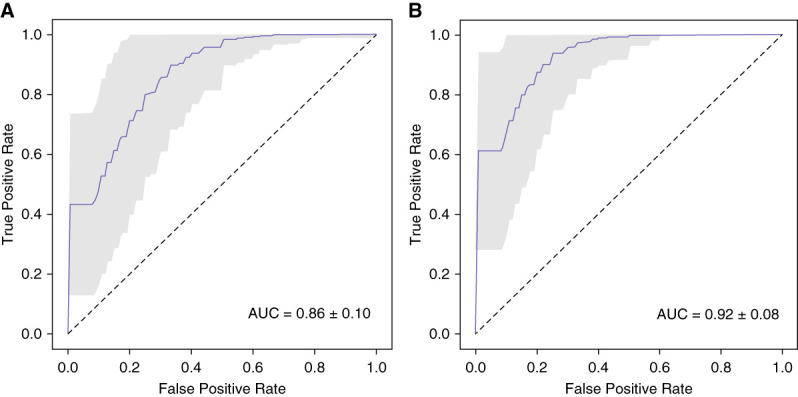
**Multispectral differentiation of exfoliated PTCs between study participants with ATN or IFTA.** (A) ROC curve for our obtained cell classifier using the optimal spectral feature combination selected by method 1. (B) ROC curve obtained using the AutoGluon classifier framework for comparative analysis (*i.e*., method 2) with the same feature combination which was selected by method 1.

On selecting their optimal feature combination (Supplemental Table 3) using method 1, we were able to categorise PTCs between these two groups with an AUC value of 0.86±0.10 (Figure 3A). The analysis using AutoGluon in method 2 and the same feature combination revealed significant differences in multispectral cellular feature patterns between exfoliated PTCs from study participants in the two groups evaluated here with an AUC value of 0.92±0.08 (Figure 3B).

### Differentiation of Multispectral Autofluorescence Signals in Exfoliated PTCs between Study Participants with Graft Rejection and IFTA

**Figure 4:**
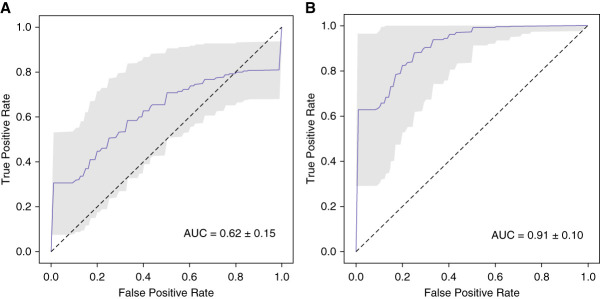
**Multispectral differentiation of exfoliated PTCs between study participants with graft rejection or IFTA.** (A) ROC curve for our obtained cell classifier using the optimal spectral feature combination selected by method 1. (B) ROC curve obtained using the AutoGluon classifier framework for comparative analysis (*i.e*., method 2) with the same feature combination which was selected by method 1.

On selecting their optimal feature combination (Supplemental Table 3) using method 1, we were able to categorise PTCs between these two groups with an AUC value of 0.62±0.15 (Figure 4A). The analysis using AutoGluon in method 2 and the same feature combination revealed significant differences in multispectral cellular features between exfoliated PTCs from study participants in the two groups investigated here with an AUC value of 0.91±0.10 (Figure 4B).

## Discussion

Our proof-of-concept study was able to demonstrate an excellent degree of discrimination between urinary exfoliated PTCs extracted from individuals with various histopathologic complications after kidney transplantation, namely, between ATN versus graft rejection, ATN versus IFTA, and graft rejection versus IFTA when using the AutoGluon software-based classifier optimisation approach, where group differences were very distinctive.

Cell exfoliation is an active biochemical process that has been linked to the homeostasis of mammalian organs.^33^ Exfoliation occurs where extracellular matrix components that usually tightly connect between cells within a structure break off, with live and dead external cells removed from the epithelial luminal surface.^34^ Cell exfoliation is thought to be under the control of cell metabolism, and the properties of kidney cells exfoliated into urine are now deemed as a potentially useful indicator of kidney pathology.^33,35^ The role of urinary exfoliated cells in the context of kidney transplantation is less well studied, but there have been reports describing the assessment of urinary exfoliated PTCs in kidney transplant recipients.^36,37^ PTCs have an important role in the histopathology of ATN, graft rejection, and IFTA, in comparison with other cell types which are exfoliated in the urine including podocytes and distal tubular cells, and hence PTCs may have greater relevance for diagnostic utility within the post-transplant setting.^13,14^ In a study involving 12 circulatory death donor kidney transplant recipients, Pizzuti *et al.*^36^ aimed to characterize the phenotype and potential applications of urine-derived renal epithelial cells (URECs) that were voided post-transplant. The URECs that are frequently obtained in the early stages after kidney transplantation have been shown to conventionally be derived from proximal tubules. Voided URECs had high proliferating and inflammatory properties, suggesting their potential role in prognosticating post-transplant ischemia-reperfusion and AKI states as well as their immunomodulatory potential. Otherwise, there was also a study by Goerlich *et al.*^37^ which aimed to determine whether the quantification of different urinary cells would allow for noninvasive detection of post-transplant graft rejection. The investigators measured urinary cell numbers of CD4^+^ and CD8^+^ T cells, monocytes/macrophages, urinary exfoliated tubular epithelial cells (TECs), and podocalyxin positive (PDX+) cells using flow cytometry and correlated their findings to biopsy reports. It was found that combining the amount of urinary T cells and TECs, or T cells and PDX+ cells, demonstrated significant differentiation between patients with graft rejection from those without (AUC 0.90 and 0.89, respectively, both *P* < 0.01). Therefore, the combination of urinary T cells and TECs or urinary T cells and PDX+ cells may be a useful resource for noninvasive detection of post-transplant graft rejection. This study suggests that urinary cell populations analyzed by flow cytometry have the potential to introduce novel noninvasive monitoring methods for kidney transplant recipients.

Innovation of novel urinary biomarkers and subsequent development of precision assessment methods to differentiate between the histopathologic causes of graft dysfunction such as ATN versus rejection over the past decade have otherwise been associated with the utilization of various molecular biomarkers such as urinary multiomics markers (*e.g*., urinary transcriptomics and proteomics) and urinary exfoliated extracellular vesicles, in which their application in post-transplant monitoring are increasingly promising.^10,11,38,39^

To the best of our knowledge, this report is the first to evaluate multispectral autofluorescence characteristics in exfoliated human transplanted cells from the kidney. Quantitative analysis of multispectral autofluorescence, which is vastly superior to subjective human inspection for reproducibility, objectivity, and rigor, has been used to differentiate between the pathophysiologic properties of transplanted cells in animal models previously. Campbell *et al.*^40^ defined an autofluorescence scoring system for ischemic pancreatic islets that accurately predicted the restoration of glucose control in diabetic mouse recipients after transplantation. With similar results being obtained for islet single-cell suspensions indicating translational utility of this novel methodology in the context of emerging beta-cell replacement strategies, these early data support the potential of multispectral imaging in prognosticating organ transplant outcomes. Ultimately, further work to validate this approach in human studies is needed, given the properties and quality of cells in mice and humans are different.

In addition to the novelty of pursuing multispectral autofluorescence analysis in urinary exfoliated PTCs, our study also contributed toward the emerging literature which studied the application of artificial intelligence machine learning–based analysis to predict causes of graft dysfunction after kidney transplantation. Since its inception in 2020 by Amazon Science, application of the AutoGluon automated machine learning framework as a tool to prognosticate the trajectory of health outcomes is emerging.^41–46^ Notably, it has been shown to be an effective tool to predict outcomes related to heart disease,^41^ diabetes,^42^ stroke (as well as to classify stroke pathology),^43,44^ and, more recently, coronavirus disease 2019 (based on analysis of multiomics).^45^ In nephrology, AutoGluon has been applied on electronic medical records data to prognosticate risk of AKI after major cardiac surgery with positive results, predicting AKI before clinical detection in 89% of cases.^46^ Within the kidney transplantation context, the use of artificial intelligence–based analysis otherwise has been mainly limited to improving radiologic and histopathologic evaluation of the allograft currently but is increasingly touted to be a useful tool in the evaluation of urinary molecular biomarkers such as multiomics markers.^47–51^ Our data suggest that AutoGluon-based analysis of urinary PTCs multispectral autofluorescence can have important implications when diagnosing patients with graft dysfunction after kidney transplantation. In scenarios where there is considerable overlap in quantitative autofluorescence features in which nonlinear feature interaction analysis is required, such as when comparing between the graft rejection versus IFTA groups in our pilot study here, AutoGluon significantly outperformed random forest classifiers for predictive accuracy.

The limitations of this study are as follows. First, owing to a small study sample size, we are currently unable to relate any changes in autofluorescence to other potential demographic and clinical factors of influence outside of the histopathologic differences classified by transplant biopsy, which limit conclusions on causal relationships that could be made based on our data alone. Putative parameters that may influence cell autofluorescence include cell aging and senescence, sex, and clinical factors such as eGFR, urinary protein, hemoglobin A1c. Furthermore, given its retrospective nature, this study is also limited in systematically addressing for the differences between the three biopsy groups in relation to time from transplant operation to biopsy. It would be expected that the time from transplant to biopsy for ATN would be significantly shorter compared with IFTA, which develops progressively, while there would be fluctuation in the time between transplant and biopsy across the rejection cohort. It is important to note that our data demonstrated significant discrimination between the groups, although it is impossible to control the timing of urine collection in our study, which depends on the timing of the transplant biopsy. Cell autofluorescence findings would be directly related to the type of post-transplant histopathology. To biologically translate the important spectral features identified from our study through spectral unmixing analysis^52^ in determining the native fluorophores which may explain the pathophysiologic processes differentiating between histopathologic causes of graft dysfunction post-transplant is a key next step where further study is required. Research on an optimal approach to perform spectral unmixing for live, unplated biologic cells is ongoing and subject to prospective study by not only our research group, but across the wider biophotonics community. Further subgroup study evaluating between individuals with graft rejection but without progressive IFTA versus those with both graft rejection and progressive IFTA and progressive IFTA only is needed. Pursuing this subgroup analysis for patients >3 months post-transplantation would be clinically relevant in particular. The rejection cohort could not be categorized into further subgroups (*e.g*., T-cell–mediated rejection versus antibody-mediated rejection) in this proof-of-concept study for meaningful analysis given the low number of study participants. We are also unable to demonstrate confirmation that all of the urinary exfoliated cells analyzed are donor derived, as we have not collected pretransplant urine samples in this proof-of-concept study. It is challenging to assess pretransplant urine samples, given most patients were oliguric or anuric with ESKD before transplantation. In our study, only two patients received preemptive transplantation in each of the ATN and the graft rejection groups, respectively, and one patient received preemptive transplantation in the IFTA group. It is probable that some of the exfoliated cells may have originated from the residual kidney of the transplant recipient. However, considering similarly small proportions of patients with preemptive kidney transplantation across the three biopsy groups, the data generated would mostly represent the state of the transplanted kidneys, as supported by the strong AUC results presented in this study.

In summary, our findings demonstrate a proof-of-concept that measurement of urinary exfoliated PTCs autofluorescence could be used to differentiate between ATN, graft rejection and IFTA in kidney transplant recipients diagnosed with these complications after transplant biopsy with an excellent degree of accuracy. Given the retrieval of human urine is noninvasive, convenient to obtain and given our methodology in extracting and assessing urinary exfoliated PTCs *via* multispectral autofluorescence imaging is robust, it is foreseeable that this technique could be implemented in post-transplant settings as a clinical decision-making tool to guide diagnosis and patient management in the future.

## Supplementary Material

## Data Availability

Anonymized data created for the study are or will be available in a persistent repository upon publication. Software Executable Code. Encoded Data. Experimental Data. Raw Data/Source Data. GitHub. Study participant-anonymized raw feature data and the computer scripts relating to the trained machine learning models used for performance evaluation presented in this study are available on GitHub (https://github.com/Aaron-lyd/CKD_detection). The raw feature data are under files “ATN-1_18042025.xlsx” (https://github.com/Aaron-lyd/CKD_detection/blob/main/Henry_2024Jan/ATN-1_18042025.xlsx) for data collected from the ATN group, “graft_rejection_0_18042025.xlsx” (https://github.com/Aaron-lyd/CKD_detection/blob/main/Henry_2024Jan/graft_rejection_0_18042025.xlsx) for data collected from the graft rejection group, and “no_rejection_1_18042025.xlsx” (https://github.com/Aaron-lyd/CKD_detection/blob/main/Henry_2024Jan/no_rejection_1_18042025.xlsx) for data collected from the IFTA group. The computer scripts relating to the trained machine learning models used for performance evaluation are under files “Henry_analysis_trans_AG.ipynb” (https://github.com/Aaron-lyd/CKD_detection/blob/main/Henry_2024Jan/Henry_analysis_trans_AG.ipynb) for the analysis between the ATN versus graft rejection group, “Henry_analysis_trans_AN.ipynb” (https://github.com/Aaron-lyd/CKD_detection/blob/main/Henry_2024Jan/Henry_analysis_trans_AN.ipynb) for the analysis between the ATN versus IFTA group, and “Henry_analysis_trans_GN.ipynb” (https://github.com/Aaron-lyd/CKD_detection/blob/main/Henry_2024Jan/Henry_analysis_trans_GN.ipynb) for the analysis between the graft rejection versus IFTA group. Further data that support the findings of this study are available from the corresponding author upon reasonable request.

## Supplemental Material

Supplemental Table 1. Demographic and clinical characteristics for individual study participants with post-transplant ATN, graft rejection and IFTA.

Supplemental Table 2. Details of the spectral channels used in this study for spectral imaging.

Supplemental Table 3. Optimal spectral feature combination selected to differentiate between urinary exfoliated PTCs in the three groups based on method.

Supplemental Figure 1. Brightfield and hyperspectral channel images from representative urinary exfoliated PTCs across the defined study groups.
