# Supplementary material for: Noninvasive Assessment of Urinary Exfoliated Proximal Tubule Cell Multispectral Autofluorescence May Differentiate between Causes of Kidney Transplant Dysfunction
Source: Kidney360. 2025 Jun 20;6(11):1853–62. doi: 10.34067/KID.0000000879 (PMC12626663; doi:10.34067/KID.0000000879)
Supplement: Supplementary file 1 [file kidney360-6-1853-s001.pdf]

ASN American Society of Nephrology
JASN Journal of the American Society of Nephrology
KIDNEY360 Accessing Our World From Every Angle
CJASN Clinical Journal of the American Society of Nephrology

ASN Journal Disclosure Form

As per ASN journal policy, I have disclosed any financial relationships or commitments I have held in the past 36 months as included below. I have listed my Current Employer below to indicate there is a relationship requiring disclosure. If no relationship exists, my Current Employer is not listed.

A. Agha reports the following:
Employer: UNSW School of Biomedical Engineering; and Ownership Interest: AMGN, PFE, JNJ, MRNA.

I understand that the information above will be published within the journal article, if accepted, and that failure to comply and/or to accurately and completely report the potential financial conflicts of interest could lead to the following: 1) Prior to publication, article rejection, or 2) Post-publication, sanctions ranging from, but not limited to, issuing a correction, reporting the inaccurate information to the authors' institution, banning authors from submitting work to ASN journals for varying lengths of time, and/or retraction of the published work.

Name: Adnan Agha
Manuscript ID: K360-2025-000163R1
Manuscript Title: Non-invasive assessment of urinary exfoliated proximal tubule cell multispectral autofluorescence may differentiate between causes of kidney transplant dysfunction
Date of Completion: May 6, 2025
Disclosure Updated Date: May 6, 2025

ASN Journal Disclosure Form

As per ASN journal policy, I have disclosed any financial relationships or commitments I have held in the past 36 months as included below. I have listed my Current Employer below to indicate there is a relationship requiring disclosure. If no relationship exists, my Current Employer is not listed.

A. Bhargava reports the following:
Employer: UNSW; and Patents or Royalties: AU2024904153A0 (Method for differentiating between histopathological causes of kidney graft dysfunction);.

I understand that the information above will be published within the journal article, if accepted, and that failure to comply and/or to accurately and completely report the potential financial conflicts of interest could lead to the following: 1) Prior to publication, article rejection, or 2) Post-publication, sanctions ranging from, but not limited to, issuing a correction, reporting the inaccurate information to the authors' institution, banning authors from submitting work to ASN journals for varying lengths of time, and/or retraction of the published work.

Name: Akanksha Bhargava
Manuscript ID: K360-2025-000163R1
Manuscript Title: Non-invasive assessment of urinary exfoliated proximal tubule cell multispectral autofluorescence may differentiate between causes of kidney transplant dysfunction
Date of Completion: June 14, 2025
Disclosure Updated Date: June 14, 2025

ASN American Society of Nephrology JASN Journal of the American Society of Nephrology KIDNEY360 Accessing Our World From Every Angle CJASN Clinical Journal of the American Society of Nephrology

ASN Journal Disclosure Form

As per ASN journal policy, I have disclosed any financial relationships or commitments I have held in the past 36 months as included below. I have listed my Current Employer below to indicate there is a relationship requiring disclosure. If no relationship exists, my Current Employer is not listed.

E. Goldys reports the following:
Employer: University of New South Wales; Ownership Interest: EosGene Therapeutics Pty; Research Funding: Biopoint Pty Ltd, Avicena Systems Pty Ltd, ConryTech Pty Ltd; and Patents or Royalties: I have granted patents that are under licence options and filed patents.

I understand that the information above will be published within the journal article, if accepted, and that failure to comply and/or to accurately and completely report the potential financial conflicts of interest could lead to the following: 1) Prior to publication, article rejection, or 2) Post-publication, sanctions ranging from, but not limited to, issuing a correction, reporting the inaccurate information to the authors' institution, banning authors from submitting work to ASN journals for varying lengths of time, and/or retraction of the published work.

Name: Ewa M. Goldys
Manuscript ID: K360-2025-000163R1
Manuscript Title: Non-invasive assessment of urinary exfoliated proximal tubule cell multispectral autofluorescence may differentiate between causes of kidney transplant dysfunction
Date of Completion: May 5, 2025
Disclosure Updated Date: May 5, 2025

ASN Journal Disclosure Form

As per ASN journal policy, I have disclosed any financial relationships or commitments I have held in the past 36 months as included below. I have listed my Current Employer below to indicate there is a relationship requiring disclosure. If no relationship exists, my Current Employer is not listed.

S. Handley reports the following:
Employer: University of New South Wales - Kensington Campus: University of New South Wales; and Patents or Royalties: AU2024904153A0 (Method for differentiating between histopathological causes of kidney graft dysfunction).

I understand that the information above will be published within the journal article, if accepted, and that failure to comply and/or to accurately and completely report the potential financial conflicts of interest could lead to the following: 1) Prior to publication, article rejection, or 2) Post-publication, sanctions ranging from, but not limited to, issuing a correction, reporting the inaccurate information to the authors' institution, banning authors from submitting work to ASN journals for varying lengths of time, and/or retraction of the published work.

Name: Shannon Handley
Manuscript ID: K360-2025-000163R1
Manuscript Title: Non-invasive assessment of urinary exfoliated proximal tubule cell multispectral autofluorescence may differentiate between causes of kidney transplant dysfunction
Date of Completion: May 8, 2025
Disclosure Updated Date: May 8, 2025

ASN
American Society of Nephrology
JASN
Journal of the American Society of Nephrology
KIDNEY360
Accessing Our World From Every Angle
CJASN
Clinical Journal of the American Society of Nephrology

ASN Journal Disclosure Form

As per ASN journal policy, I have disclosed any financial relationships or commitments I have held in the past 36 months as included below. I have listed my Current Employer below to indicate there is a relationship requiring disclosure. If no relationship exists, my Current Employer is not listed.

A. Knab reports the following:
Employer: UNSW; and Patents or Royalties: AU2024904153A0 (Method for differentiating between histopathological causes of kidney graft dysfunction).

I understand that the information above will be published within the journal article, if accepted, and that failure to comply and/or to accurately and completely report the potential financial conflicts of interest could lead to the following: 1) Prior to publication, article rejection, or 2) Post-publication, sanctions ranging from, but not limited to, issuing a correction, reporting the inaccurate information to the authors' institution, banning authors from submitting work to ASN journals for varying lengths of time, and/or retraction of the published work.

Name: Aline Knab
Manuscript ID: K360-2025-000163R1
Manuscript Title: Non-invasive assessment of urinary exfoliated proximal tubule cell multispectral autofluorescence may differentiate between causes of kidney transplant dysfunction
Date of Completion: May 5, 2025
Disclosure Updated Date: May 5, 2025

ASN
American Society of Nephrology
JASN
Journal of the American Society of Nephrology
KIDNEY360
Accessing Our World From Every Angle
CJASN
Clinical Journal of the American Society of Nephrology

ASN Journal Disclosure Form

As per ASN journal policy, I have disclosed any financial relationships or commitments I have held in the past 36 months as included below. I have listed my Current Employer below to indicate there is a relationship requiring disclosure. If no relationship exists, my Current Employer is not listed.

Y. Lang reports the following:
Employer: UNSW; and Patents or Royalties: AU2024904153A0; Method for differentiating between histopathological causes of kidney graft dysfunction;.

I understand that the information above will be published within the journal article, if accepted, and that failure to comply and/or to accurately and completely report the potential financial conflicts of interest could lead to the following: 1) Prior to publication, article rejection, or 2) Post-publication, sanctions ranging from, but not limited to, issuing a correction, reporting the inaccurate information to the authors' institution, banning authors from submitting work to ASN journals for varying lengths of time, and/or retraction of the published work.

Name: Yandong Lang
Manuscript ID: K360-2025-000163R1
Manuscript Title: Non-invasive assessment of urinary exfoliated proximal tubule cell multispectral autofluorescence may differentiate between causes of kidney transplant dysfunction
Date of Completion: May 5, 2025
Disclosure Updated Date: May 5, 2025

ASN American Society of Nephrology | JASN Journal of the American Society of Nephrology | KIDNEY360 Accessing Our World From Every Angle | CJASN Clinical Journal of the American Society of Nephrology

ASN Journal Disclosure Form

As per ASN journal policy, I have disclosed any financial relationships or commitments I have held in the past 36 months as included below. I have listed my Current Employer below to indicate there is a relationship requiring disclosure. If no relationship exists, my Current Employer is not listed.

C. Pollock reports the following:
Employer: The University of Sydney; Honoraria: Astra Zeneca, Otsuka, Vifor CSL, Boehringer Ingelheim, Eli Lily, Astellas, Glaxo-Smith Kline, Novartis, Bayer; Advisory or Leadership Role: Astra Zeneca, Janssen Cilag, Otsuka, Vifor CSL, Novartis, Boehringer Ingelheim, Pharmaxis (all paid); Certa Therapeutics, Photobionic Research Institute (Unpaid); Chair Kidney Health Australia (unpaid) and Deputy Chair Advisory Committee for the Australian Organ Transplant Authority (sitting fees. Chair NSW Bureau of Health Information (paid); Speakers Bureau: Astra Zeneca, Otsuka, Boehringher Ingelheim, Bayer; and Other Interests or Relationships: Nil apart from those listed above.

I understand that the information above will be published within the journal article, if accepted, and that failure to comply and/or to accurately and completely report the potential financial conflicts of interest could lead to the following: 1) Prior to publication, article rejection, or 2) Post-publication, sanctions ranging from, but not limited to, issuing a correction, reporting the inaccurate information to the authors' institution, banning authors from submitting work to ASN journals for varying lengths of time, and/or retraction of the published work.

Name: Carol A. Pollock
Manuscript ID: K360-2025-000163R1
Manuscript Title: Non-invasive assessment of urinary exfoliated proximal tubule cell multispectral autofluorescence may differentiate between causes of kidney transplant dysfunction.
Date of Completion: May 5, 2025
Disclosure Updated Date: October 9, 2024

ASN American Society of Nephrology
JASN Journal of the American Society of Nephrology
KIDNEY360 Accessing Our World From Every Angle
CJASN Clinical Journal of the American Society of Nephrology

ASN Journal Disclosure Form

As per ASN journal policy, I have disclosed any financial relationships or commitments I have held in the past 36 months as included below. I have listed my Current Employer below to indicate there is a relationship requiring disclosure. If no relationship exists, my Current Employer is not listed.

S. Saad reports the following:
Employer: University of Sydney; and Advisory or Leadership Role: Scientific Committee of the Australian and New Zealand Society of Nephrology; Editor in MDPI Nutrients; Guest editor BMC nephrology; Co-Chair: Cardiovascular and Renal Priority Research Area Meeting; Review panel member - Kidney Health Australia; All non-paid.

I understand that the information above will be published within the journal article, if accepted, and that failure to comply and/or to accurately and completely report the potential financial conflicts of interest could lead to the following: 1) Prior to publication, article rejection, or 2) Post-publication, sanctions ranging from, but not limited to, issuing a correction, reporting the inaccurate information to the authors' institution, banning authors from submitting work to ASN journals for varying lengths of time, and/or retraction of the published work.

Name: Sonia Saad
Manuscript ID: K360-2025-000163R1
Manuscript Title: Non-invasive assessment of urinary exfoliated proximal tubule cell multispectral autofluorescence may differentiate between causes of kidney transplant dysfunction
Date of Completion: May 5, 2025
Disclosure Updated Date: May 5, 2025

ASN American Society of Nephrology
JASN Journal of the American Society of Nephrology
KIDNEY360 Accessing Our World From Every Angle
CJASN Clinical Journal of the American Society of Nephrology

ASN Journal Disclosure Form

As per ASN journal policy, I have disclosed any financial relationships or commitments I have held in the past 36 months as included below. I have listed my Current Employer below to indicate there is a relationship requiring disclosure. If no relationship exists, my Current Employer is not listed.

Y. Tian has nothing to disclose.

I understand that the information above will be published within the journal article, if accepted, and that failure to comply and/or to accurately and completely report the potential financial conflicts of interest could lead to the following: 1) Prior to publication, article rejection, or 2) Post-publication, sanctions ranging from, but not limited to, issuing a correction, reporting the inaccurate information to the authors' institution, banning authors from submitting work to ASN journals for varying lengths of time, and/or retraction of the published work.

Name: Yuan Tian
Manuscript ID: K360-2025-000163R1
Manuscript Title: Non-invasive assessment of urinary exfoliated proximal tubule cell multispectral autofluorescence may differentiate between causes of kidney transplant dysfunction
Date of Completion: May 6, 2025
Disclosure Updated Date: May 6, 2025

ASN
American Society of Nephrology
JASN
Journal of the American Society of Nephrology
KIDNEY360
Accessing Our World From Every Angle
CJASN
Clinical Journal of the American Society of Nephrology

ASN Journal Disclosure Form

As per ASN journal policy, I have disclosed any financial relationships or commitments I have held in the past 36 months as included below. I have listed my Current Employer below to indicate there is a relationship requiring disclosure. If no relationship exists, my Current Employer is not listed.

H. Wu reports the following:
Employer: Kolling Institute of Medical Research, The University of Sydney; Research Funding: Polycystic Kidney Disease Australia; Patents or Royalties: Method for differentiating between histopathological causes of kidney graft dysfunction (AU2024904153A0) (IP Australia); and Advisory or Leadership Role: Non-paid member of the International Society of Nephrology CardioRenal Toolkit Workgroup; Non-paid Editorial Board Member of Frontiers in Medicine, PloS One and BMC Nephrology.

I understand that the information above will be published within the journal article, if accepted, and that failure to comply and/or to accurately and completely report the potential financial conflicts of interest could lead to the following: 1) Prior to publication, article rejection, or 2) Post-publication, sanctions ranging from, but not limited to, issuing a correction, reporting the inaccurate information to the authors' institution, banning authors from submitting work to ASN journals for varying lengths of time, and/or retraction of the published work.

Name: Henry Wu
Manuscript ID: K360-2025-000163R1
Manuscript Title: Non-invasive assessment of urinary exfoliated proximal tubule cell multispectral autofluorescence may differentiate between causes of kidney transplant dysfunction
Date of Completion: May 5, 2025
Disclosure Updated Date: May 5, 2025
