# Supplementary material for: Noninvasive Assessment of Urinary Exfoliated Proximal Tubule Cell Multispectral Autofluorescence May Differentiate between Causes of Kidney Transplant Dysfunction
Source: Kidney360. 2025 Jun 20;6(11):1853–62. doi: 10.34067/KID.0000000879 (PMC12626663; doi:10.34067/KID.0000000879)

**Supplemental Document Table of Contents**

Supplemental Table 1……………………………………………………………………………………………………..page 2

Supplemental Table 2……………………………………………………………………………………………………..page 4

Supplemental Table 3……………………………………………………………………………………………………..page 5

Supplemental Figure 1……………………………………………………………………………………………………page 8

**Supplemental Table 1.** Demographic and clinical characteristics for individual study participants with post-transplant acute tubular necrosis, graft rejection & interstitial fibrosis and tubular atrophy

| | Exp ID | Sex | Age (years) | Number of days between transplant and allograft biopsy | Diabetes mellitus status | Glycosuria | eGFR (ml/min/1.73m$^2$) | ACR | Albuminuria status | Serum creatinine (µmol/L) | Allograft biopsy diagnosis | Organ Donor sex | Organ donor age (years) | Organ donor diabetes mellitus status | Organ donor albuminuria status |
|---|---|---|---|---|---|---|---|---|---|---|---|---|---|---|---|
| **ATN** | 1 | M | 53 | 7 | NDM | No | 34 | 30 | Micro | 188 | ATN | M | 45 | NDM | No albuminuria |
| | 2 | F | 67 | 6 | NDM | No | 40 | 50 | Micro | 121 | ATN | F | 42 | NDM | No albuminuria |
| | 3 | F | 66 | 3 | NDM | No | 33 | 14 | Micro | 142 | ATN | F | 40 | NDM | No albuminuria |
| | 4 | M | 58 | 5 | T2DM | No | 6 | 45 | Micro | 813 | ATN | M | 58 | NDM | No albuminuria |
| | 5 | F | 44 | 4 | NDM | No | 21 | 268 | Macro | 238 | ATN | M | 67 | NDM | No albuminuria |
| | 6 | M | 53 | 3 | NDM | No | 55 | 30 | Micro | 126 | ATN | F | 38 | NDM | No albuminuria |
| | 7 | M | 59 | 5 | NDM | No | 39 | 333 | Macro | 164 | ATN | M | 61 | NDM | No albuminuria |
| | 8 | M | 41 | 2 | T2DM | No | 12 | 50 | Micro | 479 | ATN | F | 53 | NDM | No albuminuria |
| | 9 | M | 57 | 6 | NDM | No | 38 | 10 | Micro | 171 | ATN | F | 68 | NDM | No albuminuria |
| | 10 | M | 52 | 7 | T2DM | No | 18 | 45 | Micro | 331 | ATN | M | 41 | NDM | No albuminuria |
| **Graft Rejection** | 1 | M | 38 | 10 | NDM | No | 27 | 16 | Micro | 248 | Acute ABMR | F | 33 | NDM | No albuminuria |
| | 2 | M | 52 | 6 | NDM | No | 26 | 50 | Micro | 240 | Acute ABMR | M | 56 | NDM | No albuminuria |
| | 3 | M | 52 | 10 | NDM | No | 35 | 22 | Micro | 184 | Acute TCR | F | 61 | NDM | No albuminuria |
| | 4 | M | 26 | 153 | NDM | No | 36 | 24 | Micro | 209 | Chronic active ABMR | M | 26 | NDM | No albuminuria |
| | 5 | F | 50 | 749 | NDM | No | 27 | 427 | Macro | 185 | Chronic active ABMR | F | 65 | NDM | No albuminuria |
| | 6 | M | 35 | 172 | NDM | No | 39 | 54 | Micro | 187 | Recurrent ABMR | M | 43 | NDM | No albuminuria |
| | 7 | M | 34 | 90 | NDM | No | 49 | 12 | Micro | 157 | Chronic TCR | F | 28 | NDM | No albuminuria |

| | | | | | | | | | | | | | | | |
|---|---|---|---|---|---|---|---|---|---|---|---|---|---|---|---|
| | 8 | M | 38 | 4449 | NDM | No | 33 | 17 | Micro | 213 | Chronic TCR | M | 45 | NDM | No albuminuria |
| | 9 | F | 28 | 102 | NDM | No | 65 | 50 | Micro | 101 | Chronic TCR | F | 41 | NDM | No albuminuria |
| | 10 | M | 38 | 89 | NDM | No | 34 | 50 | Micro | 205 | Mixed chronic BCR and TCR | F | 40 | NDM | No albuminuria |
| **IFTA** | 1 | F | 43 | 2098 | NDM | No | 25 | 99 | Macro | 203 | Chronic allograft nephropathy | M | 39 | NDM | No albuminuria |
| | 2 | M | 56 | 3252 | NDM | No | 13 | 135 | Macro | 400 | Chronic allograft nephropathy (with secondary FSGS) | F | 47 | NDM | No albuminuria |
| | 3 | F | 63 | 1472 | NDM | No | 28 | 50 | Micro | 167 | CNI toxicity-associated IFTA | M | 35 | NDM | No albuminuria |
| | 4 | M | 62 | 2537 | NDM | No | 21 | 109 | Macro | 267 | CNI toxicity-associated IFTA | M | 60 | NDM | No albuminuria |
| | 5 | F | 27 | 1780 | NDM | No | 23 | 65 | Micro | 249 | CNI toxicity-associated IFTA | F | 32 | NDM | No albuminuria |
| | 6 | F | 55 | 854 | NDM | No | 28 | 500 | Macro | 238 | CNI toxicity-associated IFTA | M | 64 | NDM | No albuminuria |
| | 7 | M | 55 | 4941 | T2DM | No | 59 | 59 | Micro | 119 | DM nephropathy | F | 40 | NDM | No albuminuria |
| | 8 | F | 45 | 767 | NDM | No | 28 | 53 | Micro | 185 | Progressive fibrosis in the absence of immunological challenge | F | 39 | NDM | No albuminuria |
| | 9 | M | 60 | 2457 | NDM | No | 20 | 468 | Macro | 287 | Progressive fibrosis in the absence of immunological challenge | M | 52 | NDM | No albuminuria |
| | 10 | M | 75 | 843 | NDM | No | 39 | 50 | Micro | 150 | Progressive fibrosis in the absence of immunological challenge | F | 58 | NDM | No albuminuria |

ABMR: Antibody-mediated rejection; ACR: Albumin creatinine ratio; ATN: Acute tubular necrosis; BCR: B-cell rejection; CNI: Calcineurin inhibitor; DM: Diabetes mellitus; eGFR: Estimated glomerular filtration rate; F: Female; FSGS: Focal segmental glomerular sclerosis; ID: Identification; IFTA: Interstitial fibrosis and tubular atrophy; M: Male; Macro: Macroalbuminuria; Micro: Microalbuminuria; NDM: No diabetes mellitus; TCR: T-cell rejection; T2DM: Type 2 diabetes mellitus

**Supplemental Table 2.** Details of the spectral channels used in this study for spectral imaging

| Spectral Channels Number | Excitation wavelength (nm) | Emission wavelength (bandwidth) (nm) | Dichroic mirror longpass (nm) | Exposure (sec) | EM gain | Number of image acquisitions to calculate 'averages' | Power at objective (µW) |
|---|---|---|---|---|---|---|---|
| 1 | 345 | 391-437 | 389 | 5 | 1 | 3 | 2.8 |
| 2 | 345 | 398-504 | 389 | 5 | 1 | 3 | 2.9 |
| 3 | 345 | 545-604 | 552 | 5 | 1 | 3 | 2.7 |
| 4 | 490 | 545-604 | 552 | 5 | 1 | 3 | 4.0 |
| 5 | 505 | 545-604 | 552 | 5 | 1 | 3 | 8.4 |
| 6 | 345 | 611-900 | 552 | 5 | 1 | 3 | 2.9 |
| 7 | 490 | 611-900 | 552 | 5 | 1 | 3 | 4.1 |
| 8 | 505 | 611-900 | 552 | 5 | 1 | 3 | 8.6 |
| 9 | 358 | 391-437 | 389 | 5 | 1 | 3 | 2.8 |
| 10 | 371 | 391-437 | 389 | 5 | 1 | 3 | 3.1 |
| 11 | 377 | 391-437 | 389 | 5 | 1 | 3 | 2.1 |
| 12 | 371 | 398-504 | 389 | 5 | 1 | 3 | 3.1 |
| 13 | 377 | 398-504 | 389 | 5 | 1 | 3 | 2.2 |
| 14 | 381 | 398-504 | 389 | 5 | 1 | 3 | 0.9 |
| 15 | 358 | 545-604 | 552 | 5 | 1 | 3 | 3.0 |
| 16 | 371 | 545-604 | 552 | 5 | 1 | 3 | 4.6 |
| 17 | 377 | 545-604 | 552 | 5 | 1 | 3 | 8.5 |

| | | | | | | | |
|---|---|---|---|---|---|---|---|
| 18 | 381 | 545-604 | 552 | 5 | 1 | 3 | 10.2 |
| 19 | 391 | 545-604 | 552 | 5 | 1 | 3 | 6.9 |
| 20 | 397 | 545-604 | 552 | 5 | 1 | 3 | 8.5 |
| 21 | 400 | 545-604 | 552 | 5 | 1 | 3 | 9.2 |
| 22 | 403 | 545-604 | 552 | 5 | 1 | 3 | 4.9 |
| 23 | 406 | 545-604 | 552 | 5 | 1 | 3 | 9.7 |
| 24 | 412 | 545-604 | 552 | 5 | 1 | 3 | 13.5 |
| 25 | 437 | 545-604 | 552 | 5 | 1 | 3 | 14.3 |
| 26 | 457 | 545-604 | 552 | 5 | 1 | 3 | 8.3 |
| 27 | 406 | 611-900 | 552 | 5 | 1 | 3 | 10.2 |
| 28 | 412 | 611-900 | 552 | 5 | 1 | 3 | 14.3 |
| 29 | 418 | 611-900 | 552 | 5 | 1 | 3 | 15.5 |
| 30 | 430 | 611-900 | 552 | 5 | 1 | 3 | 10.7 |
| 31 | 437 | 611-900 | 552 | 5 | 1 | 3 | 14.9 |
| 32 | 457 | 611-900 | 552 | 5 | 1 | 3 | 8.6 |
| 33 | 469 | 611-900 | 552 | 5 | 1 | 3 | 12.3 |
| 34 | 476 | 611-900 | 552 | 5 | 1 | 3 | 13.4 |
| 35 | 476 | 545-604 | 552 | 0.01 | 1 | 1 | - |

EM: Electron-multiplying gain

**Supplemental Table 3.** Optimal spectral feature combination selected to differentiate between urinary exfoliated proximal tubule cells in the three groups based on method **(i)**

| |
|---|
| *Optimal feature combination selected when evaluating between urinary exfoliated PTCs from study participants with ATN versus graft rejection* |
| Ratio of mean value of channel 8 and mean value of top 10% of channel 2 |
| Ratio of mean value of top 10% of channel 31 and mean value of top 10% of channel 32 |

| |
|---|
| Ratio of mean value of top 10% of channel 24 and mean value of top 10% of channel 28 |
| Ratio of mean value of top 10% of channel 26 and mean value of top 10% of channel 23 |
| Ratio of mean value of channel 22 to mean value of top 40% of channel 12 |
| Ratio of mean value of channel 27 to mean value of top 40% of channel 5 |
| Ratio of mean value of channel 21 to mean value of top 40% of channel 25 |
| Ratio of mean value of channel 24 to mean value of top 40% of channel 7 |
| *Optimal feature combination selected when evaluating between urinary exfoliated PTCs from study participants with ATN versus IFTA* |
| Ratio of mean value of top 10% of channel 24 and mean value of top 10% of channel 28 |
| Ratio of mean value of top 10% of channel 26 and mean value of top 10% of channel 23 |
| Ratio of mean value of top 10% of channel 26 and mean value of top 10% of channel 21 |
| Ratio of mean value of channel 5 to mean value of top 40% of channel 4 |
| Ratio of mean value of channel 22 to mean value of top 40% of channel 12 |
| Ratio of mean value of channel 21 to mean value of top 40% of channel 3 |
| Ratio of mean value of channel 22 to mean value of top 40% of channel 15 |
| Ratio of mean value of channel 21 to mean value of top 40% of channel 25 |

| |
|---|
| *Optimal feature combination selected when evaluating between urinary exfoliated PTCs from study participants with graft rejection versus IFTA* |
| Ratio of mean value of top 10% of channel 2 and mean value of top 10% of channel 5 |
| Ratio of mean value of top 10% of channel 26 and mean value of top 10% of channel 27 |
| Ratio of mean value of channel 18 and mean value of top 10% of channel 3 |
| Ratio of mean value of top 10% of channel 5 and mean value of top 10% of channel 23 |
| Ratio of mean value of top 10% of channel 31 and mean value of top 10% of channel 18 |
| Ratio of mean value of channel 8 to mean value of top 40% of channel 21 |
| Ratio of mean value of channel 5 to mean value of top 40% of channel 4 |
| Ratio of mean value of channel 29 to mean value of top 40% of channel 2 |

ATN: Acute tubular necrosis; IFTA: Interstitial fibrosis and tubular atrophy; PTC: Proximal tubule cells

**Supplemental Figure 1.** Brightfield and hyperspectral (HS) channel images from representative urinary exfoliated proximal tubule cells across the defined study groups

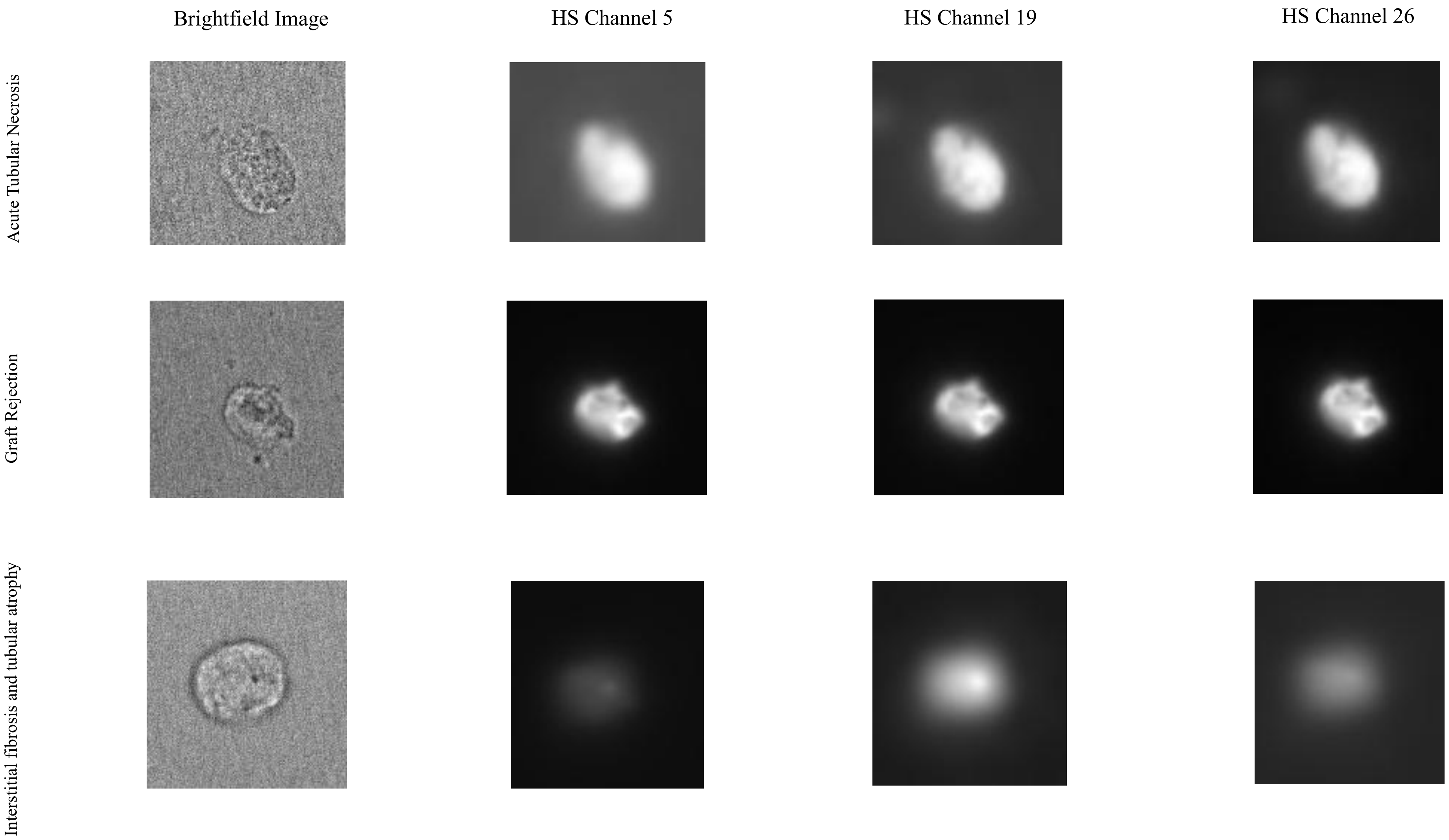

Supplement: Supplementary file 2 [file kidney360-6-1853-s002.pdf]
